# Supplementary material for: Rapid electrochemical detection of coronavirus SARS-CoV-2
Source: Nat Commun. 2021 Feb 5;12:802. doi: 10.1038/s41467-021-21121-7 (PMC7864991; doi:10.1038/s41467-021-21121-7)
Supplement: Supplementary file 1 — Supplementary Information [file 41467_2021_21121_MOESM1_ESM.pdf]

Supplementary Information
Rapid Electrochemical Detection of Coronavirus SARS-CoV-2.

Thanyarat Chaibun[1], Jiratchaya Puenpa[2], Tatchanun Ngamdee[3], Nimaradee Boonapatcharoen[4], Pornpat Athamanolap[1], Anthony Peter O'Mullane[5], Sompong Vongpunsawad[2], Yong Poovorawan[2], Lee Su Yin[6,7], Benchaporn Lertanantawong[1*]

[*]Correspondence: Benchaporn Lertanantawong (E-mail: benchaporn.ler@mahidol.ac.th)

The PDF file includes

Supplementary Figures

Supplementary Fig. 1 The electrophoresis result of circular DNA templates with/without target gene amplificated by RCA reaction.
Supplementary Fig. 2 Electrochemical property of silica-redox dye.
Supplementary Fig. 3 Electrochemical detection in clinical samples using differential pulse voltammetry (DPV).

Supplementary Table

Supplementary Table 1 Plasmid concentrations in molarity unit.

Supplementary Figures

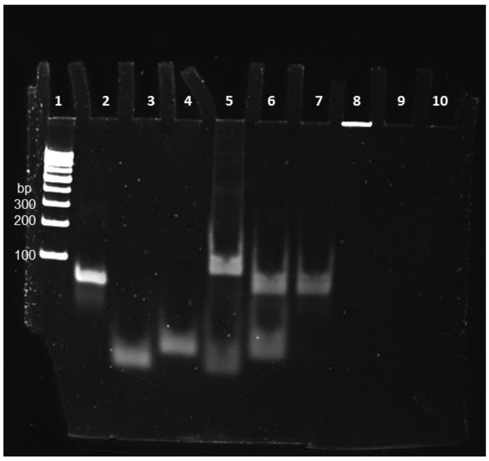

Lane 1 – Ladder 100 bp
Lane 2 - Circular DNA template for S gene
Lane 3 - DNA linear target for S genes
Lane 4 - DNA linear target for N genes
Lane 5 – Sg Circular DNA template with Sg-LT
Lane 6 – Sg Circular DNA template with non-complementary (NC)
Lane 7 – Sg Circular DNA template with phosphate buffer (PB)
Lane 8 – RCA with Padlock DNA reaction from Lane 5
Lane 9 – RCA with reaction from Lane 6
Lane 10 – RCA with reaction from Lane 7

Supplementary Fig. 1 **The electrophoresis result of circular DNA templates with/without target *S* gene amplified by RCA reaction.**

Supplementary Figure 1 shows the gel image that visualized the RCA reaction for the *S* gene (n = 3). In Lane 2, a band of the circular DNA template (66 bases) can be seen below the 100 bp band. Lanes 3 and 4 show the bands of Sg and Ng DNA linear targets. Lane 5 shows the Padlock DNA band around 100 bp, which confirmed the hybridization of circular DNA template and its target gene (*S* gene). Although Padlock DNA and circular DNA template are both 66 bases, the circular form of the Padlock DNA causes it to migrate slower than the "linear" circular DNA (Lane 2). Lane 6 and 7 show bands with the same size as circular DNA template (Lane 2) because in the absence of Sg-LT, the Padlock DNA does not form. Lane 8 shows a band at the well corresponding to the high molecular weight RCA amplicons, which confirmed the specific RCA reaction. Lanes 9 and 10 do not show any band as no RCA reaction / amplicons were formed.

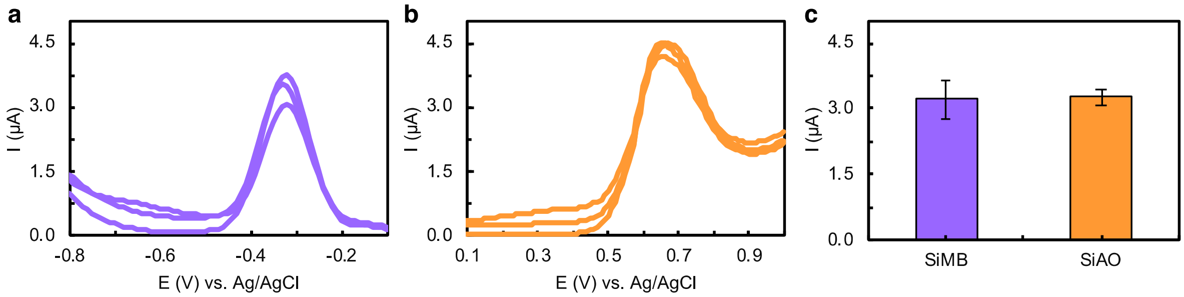

Supplementary Fig. 2 **Electrochemical property of silica-redox dye**. **a** Differential pulse voltammograms showing the current signal of SiMB (purple line) and **b** SiAO (orange line). **c** bar graphs represent current signal of both SiMB and SiAO (n=3).

Supplementary Fig. 2 shows the current signal from the oxidation of redox dyes labelled on DNA reporter probes. The silica (Si)-redox dye had been prepared separately for each dye (methylene blue (MB) and acridine orange (AO)) using the same concentration of redox dye, which is 15 µM on 0.3 g of silica. Thermodynamic and chemical interactions of these 2 silica dyes should be the same[1, 2]. Differential pulse voltammetry (DPV) was used to measure the current response from Si-MB and Si-AO at the same concentration, with 3 replicates for each dye.

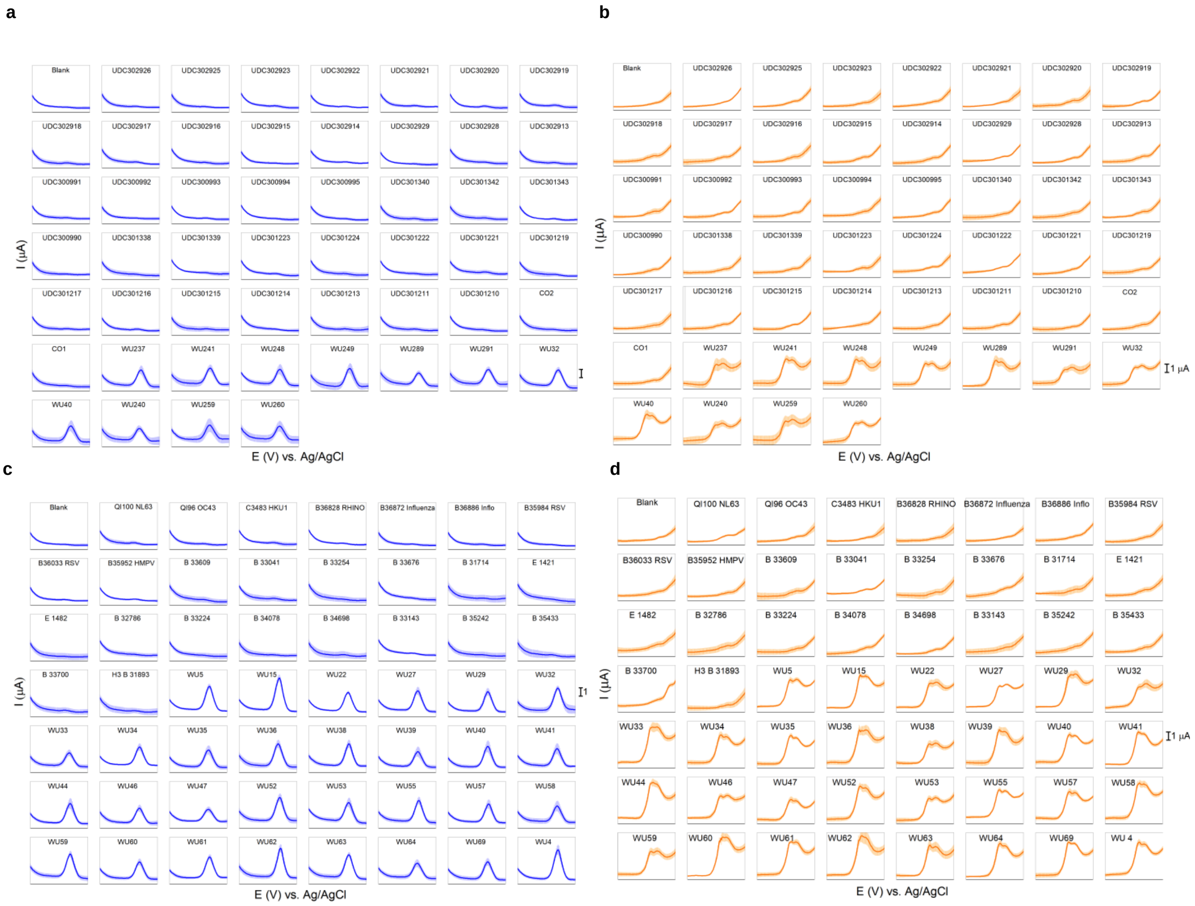

Supplementary Fig. 3 **Electrochemical detection in clinical samples using differential pulse voltammetry (DPV).** The voltammograms of SARS-CoV-2 RNA of *N*-gene (a), *S*-gene (b), and SARS-CoV-2 cDNA of *N*-gene (c) and *S*-gene (d).

Supplementary Table

Supplementary Table 1 **Plasmid concentrations in molarity unit**

Plasmid concentrations		
(copies/µL)	molarity (M)	
	N gene size 4.032 kb	*S* gene size 4.133 kb
1.00×10^{-1}	1.70×10^{-19}	1.68×10^{-19}
1.00×10^{0}	1.70×10^{-18}	1.68×10^{-18}
1.00×10^{1}	1.71×10^{-17}	1.68×10^{-17}
1.00×10^{2}	1.66×10^{-16}	1.66×10^{-16}
1.00×10^{3}	1.66×10^{-15}	1.66×10^{-15}
1.00×10^{4}	1.66×10^{-14}	1.66×10^{-14}
1.00×10^{5}	1.66×10^{-13}	1.66×10^{-13}
1.00×10^{6}	1.66×10^{-12}	1.66×10^{-12}
1.00×10^{7}	1.66×10^{-11}	1.66×10^{-11}
1.00×10^{8}	1.66×10^{-10}	1.66×10^{-10}
1.00×10^{9}	1.66×10^{-9}	1.66×10^{-9}
1.00×10^{10}	1.66×10^{-8}	1.66×10^{-8}

Supplementary References

1. Cheeveewattanagul N, Rijiravanich P, Surareungchai W, Somasundrum M. Loading of silicon nanoparticle labels with redox mediators for detection of multiple DNA targets within a single voltammetric sweep. *Journal of Electroanalytical Chemistry* 779, 61-66 (2016).

2. Palit D, Moulik SP. Adsorption of the Dyes (Methylene Blue and Acridine Orange and Their Mixtures) from Aqueous Solutions on Cholesterol Surface. *Colloid Journal* 65, 350-357 (2003).
