# Supplementary material for: Rapid electrochemical detection of coronavirus SARS-CoV-2
Source: Nat Commun. 2021 Feb 5;12:802. doi: 10.1038/s41467-021-21121-7 (PMC7864991; doi:10.1038/s41467-021-21121-7)
Supplement: Supplementary file 3 — Reporting Summary [file 41467_2021_21121_MOESM3_ESM.pdf]

nature research

Corresponding author(s): Benchaporn Lertanantawong

Last updated by author(s): Jan 5, 2021

Reporting Summary

Nature Research wishes to improve the reproducibility of the work that we publish. This form provides structure for consistency and transparency in reporting. For further information on Nature Research policies, see our Editorial Policies and the Editorial Policy Checklist.

Statistics

For all statistical analyses, confirm that the following items are present in the figure legend, table legend, main text, or Methods section.

n/a	Confirmed	
☐	☒	The exact sample size (n) for each experimental group/condition, given as a discrete number and unit of measurement
☐	☒	A statement on whether measurements were taken from distinct samples or whether the same sample was measured repeatedly
☐	☒	The statistical test(s) used AND whether they are one- or two-sided<br>*Only common tests should be described solely by name; describe more complex techniques in the Methods section.*
☒	☐	A description of all covariates tested
☒	☐	A description of any assumptions or corrections, such as tests of normality and adjustment for multiple comparisons
☐	☒	A full description of the statistical parameters including central tendency (e.g. means) or other basic estimates (e.g. regression coefficient) AND variation (e.g. standard deviation) or associated estimates of uncertainty (e.g. confidence intervals)
☐	☒	For null hypothesis testing, the test statistic (e.g. F, t, r) with confidence intervals, effect sizes, degrees of freedom and P value noted<br>*Give P values as exact values whenever suitable.*
☒	☐	For Bayesian analysis, information on the choice of priors and Markov chain Monte Carlo settings
☒	☐	For hierarchical and complex designs, identification of the appropriate level for tests and full reporting of outcomes
☒	☐	Estimates of effect sizes (e.g. Cohen's d, Pearson's r), indicating how they were calculated

Our web collection on statistics for biologists contains articles on many of the points above.

Software and code

Policy information about availability of computer code

Data collection	Data from the electrochemical measurements was collected by Palmsens4 potentiostat instrument connected to a PSTrace 5.7 software (Palmsens, The Netherlands).
Data analysis	Microsoft Excel version 16.30 was used to perform two-tailed Student's t-test for statistical analysis.

For manuscripts utilizing custom algorithms or software that are central to the research but not yet described in published literature, software must be made available to editors and reviewers. We strongly encourage code deposition in a community repository (e.g. GitHub). See the Nature Research guidelines for submitting code & software for further information.

Data

Policy information about availability of data

All manuscripts must include a data availability statement. This statement should provide the following information, where applicable:

- Accession codes, unique identifiers, or web links for publicly available datasets
- A list of figures that have associated raw data
- A description of any restrictions on data availability

The authors declare that the data supporting the findings of this study are available within the paper and its supplementary information files or from the corresponding author upon reasonable request. Sequence information used in this study was from National Center for Biotechnology Information (NCBI) with GenBank accessions of MN908947.3 (https://www.ncbi.nlm.nih.gov/). The source data involving silica particle size, current signal, voltammogram, cq-value in Figs.1 a, b, c, 2b, c, d, e, g, and 3,a,b,c,d, Supplementary Fig.1 and 2 and Supplementary Table 1 are provided as a Source Data file of this paper.

Field-specific reporting

Please select the one below that is the best fit for your research. If you are not sure, read the appropriate sections before making your selection.

☒ Life sciences ☐ Behavioural & social sciences ☐ Ecological, evolutionary & environmental sciences

For a reference copy of the document with all sections, see nature.com/documents/nr-reporting-summary-flat.pdf

Life sciences study design

All studies must disclose on these points even when the disclosure is negative.

Sample size	During the period of the study, which was the first wave of outbreak in Thailand from February to April 2020, we managed to collect a total of 106 clinical samples. From April 2020 onwards, there was a decline in the number of cases in Thailand. Due to the urgency of the situation, we included the samples that we managed to collect within the mentioned time frame of the first outbreak.<br>Sample size calculation was not performed since the aim of the study was to demonstrate the feasibility of the assay for testing with clinical samples. We benchmarked the number of samples used in this study with similar papers published recently in peer-reviewed journals [1, 2] and we found that our sample size is comparable or larger than those studies. In total, we tested 106 clinical samples.<br><br>[1] Broughton JP, et al. CRISPR–Cas12-based detection of SARS-CoV-2. Nature Biotechnology 38, 870-874 (2020).<br>[2] Ding X, et al. Ultrasensitive and visual detection of SARS-CoV-2 using all-in-one dual CRISPR-Cas12a assay. Nature Communications 11, 4711 (2020).
Data exclusions	No data exclusions
Replication	For all electrochemical measurements, five replications for independent experiments were carried out to make sure the detection results were reliable.
Randomization	The samples for specificity testing were randomized. The testing of the clinical samples was performed in a randomized manner.
Blinding	Clinical samples were tested by an individual who was blinded to RT-PCR results.

Reporting for specific materials, systems and methods

We require information from authors about some types of materials, experimental systems and methods used in many studies. Here, indicate whether each material, system or method listed is relevant to your study. If you are not sure if a list item applies to your research, read the appropriate section before selecting a response.

Materials & experimental systems

n/a	Involved in the study
☒	☐ Antibodies
☒	☐ Eukaryotic cell lines
☒	☐ Palaeontology and archaeology
☒	☐ Animals and other organisms
☒	☐ Human research participants
☐	☒ Clinical data
☒	☐ Dual use research of concern

Methods

n/a	Involved in the study
☒	☐ ChIP-seq
☒	☐ Flow cytometry
☒	☐ MRI-based neuroimaging

Clinical data

Policy information about clinical studies

All manuscripts should comply with the ICMJE guidelines for publication of clinical research and a completed CONSORT checklist must be included with all submissions.

Clinical trial registration	The research proposal was approved by the institutional review board of the Ethics Committee of the Faculty of Medicine, Chulalongkorn University, Thailand (IRB number 301/63)
Study protocol	*Note where the full trial protocol can be accessed OR if not available, explain why.*
Data collection	*Describe the settings and locales of data collection, noting the time periods of recruitment and data collection.*
Outcomes	*Describe how you pre-defined primary and secondary outcome measures and how you assessed these measures.*
